# Optimization of measurement-based care (OMBC) for depression based on all-round and continuous assessment: rationale and protocol for a multicenter randomized control clinical trial

**DOI:** 10.1186/s13063-022-06295-9

**Published:** 2022-05-03

**Authors:** Jingjing Zhou, Xiao Wang, Jian Yang, Xuequan Zhu, Le Xiao, Lei Feng, Gang Wang

**Affiliations:** 1 The National Clinical Research Center for Mental Disorders & Beijing Key Laboratory of Mental Disorders & Beijing Anding Hospital, Capital Medical University, 5 Ankang Lane, Dewai Avenue, Xicheng District, Beijing, 100088 China; 2 Advanced Innovation Center for Human Brain Protection, Capital Medical University, Beijing, China

**Keywords:** Depression, Measurement-based care, Optimization, Recurrence, Randomized controlled trial

## Abstract

**Background:**

Despite the recent findings presenting the benefits of measurement-based care (MBC) compared to treatment as usual (TAU), MBC is still not the standard of care used in clinical settings. The aim of the present study was to achieve the optimization of MBC (OMBC) for major depressive disorder (MDD) by establishing a comprehensive MBC framework based on all-round, continuous assessment for depression.

**Methods:**

The target recruitment size is 900 patients, and the study is conducted at 8 centers in China. The patients are randomly assigned to the MBC and TAU groups at a 2:1 ratio. The subjects are scheduled to remain for 12 weeks in the acute phase and for 12 months in the maintenance phase. The primary outcomes are the complete remission rate and the proportion of patients with a 16-item Quick Inventory of Depressive Symptomatology–Self-Report (QIDS-SR 16) total score ≤ 5 of the MBC and TAU groups at the acute phase, and the recurrence rate/time between the two groups is measured at the maintenance phase. Secondary outcomes included the changes in the parameters QIDS-SR 16, Patient Health Questionnaire-9 (PHQ-9), and 17-item Hamilton Rating Scale for Depression (HAMD-17) from baseline and the response rate between the two groups at the acute phase as well as the comparison of recurrence rate between the two groups at the end of the study.

**Trial registration:**

Chinese Clinical Trial Registry, ChiCTR-OOC-17012566. The registration was performed retrospectively on 4 September 2017.

## Introduction

Recent epidemiological data have reported that in China, the weighted lifetime prevalence of major depressive disorder (MDD) is approximately 3.4%, and the weighted 12­month prevalence of this disorder is estimated at 2.1% [1]. Treatment of depression is essential since it is one of the risk factors of suicide, accounting for up to 60% of suicide incidents [2–4]. Antidepressant medication is the mainstream treatment for MDD; the inconsistency of treatment strategies among clinicians has created considerable variability in practice, and the experience-based strategy (which is not based on the objective and quantitative assessment) to treat depression restricts the development of standardized treatment [5, 6]. Moreover, measurement-based care (MBC) is a relatively simple evidence-based intervention framework. The American Psychiatric Association (APA) Practice Guidelines recommend that MBC should be used to monitor the progression of depression as well as its symptom severity, treatment tolerability, and treatment safety [7].

MBC is defined as the routine measurement of symptoms and side effects at each treatment visit and the use of a treatment manual describing when and how to modify medication doses according to these measurements [8]. The MBC strategy in psychiatry includes routine assessments and the use of assessments in decision-making, suggesting that the adjustment of the dosage, treatment steps, or phases does not only rely on the clinicians’ experience [9, 10]. Preliminary studies have shown that MBC is a framework for guiding practice and can provide optimal treatment outcomes to patients compared to usual care [11]. Previous studies performed over the past 20 years indicated that MBC improved the quality of patient care [12]. Therefore, the integration of MBC into standard care has been given careful consideration.

Considering the management of depression involves comprehensive assessment, there are 3 phases in the treatment of depression—the acute phase, the continuation phase, and the maintenance phase [13]. The acute phase involves the stabilization of acute symptoms, the purpose of the continuation phase is to prevent a relapse, and the goal of maintenance therapy is to prevent recurrence [14]. However, a substantial proportion of patients with MDD experience disease relapse or recurrence (R/R), within 10 years after the first depressive episode; rates of recurrence have been reported to be as high as 85% [15, 16]. Measurement-based care (MBC) is a relatively simple self-report-based intervention framework, and using this strategy of measurement-based care with treatment algorithms may result in more patients being able to achieve remission, which is the standard goal of acute treatment [17]. MBC improves patient behavior which is related to the cognizance of the warning signs of relapse or reoccurrence in patients with major depressive disorder [18]. Although MBC exhibits benefits over usual care, it is still not the standard care in psychiatric practices, and it also needs to be improved and popularized combined with the all-round intervention of depression.

MBC was introduced in the Beijing Anding Hospital for the first time in China, and it has markedly increased the speed of achieving patient response and remission [19]. However, the follow-up period of the study was too short (24 weeks), and the recruitment protocol was not complex (only Beijing Anding Hospital) and failed to build a convenient and continuous evaluation system, which limited the promotion of MBC in China. Therefore, optimization of MBC (OMBC) treatment in the future requires immediate attention, to establish a comprehensive MBC framework based on all-round, continuous assessment for depression and contribute to maximize the therapeutic benefit and facilitate the implementation of MBC.

The present study aims to compare the efficacy and acceptability between the MBC group and the TAU group during the acute phase and further explore whether the intervention of the comprehensive intervention group in the maintenance phase will contribute to reduce the recurrence rate of patience.

## Methods

### Study design

This is a multicenter randomized control clinical trial, difference analysis, consisting of two phases, the acute phase and maintenance phase. The acute phase includes a 12-week, randomized control trial in which participants diagnosed with MDD are allocated to either the MBC or the TAU group. The subjects of both groups are administered either escitalopram (Lexapro, Xi’an Janssen Pharmaceutica) or duloxetine (Cymbalta, Eli Lilly and Company), and the MBC group includes an adjustment in the treatment plan according to the quantitative program, which is based on the self-assessment results of the subjects. The patients in the TAU group receive conventional clinical treatment during the acute phase. In the maintenance phase, the patients of the MBC treatment group who achieve a score of ≤ 9 in the 16-item Quick Inventory of Depressive Symptomatology–Self-Report (QIDS-SR 16) at the acute phase are randomly assigned to either the comprehensive intervention group or the drug treatment group for a 6-month intervention phase and a follow-up period of 12 months. The present study was retrospectively registered in the Chinese Clinical Trial Registry (ChiCTR-OOC-17012566) on 4 September 2017. The study is conducted in accordance with the “Declaration of Helsinki” and was approved by the Human Research Ethics Committees corresponding to each study site.

### Participants

The participants are recruited from depressed patients who are treated at eight psychiatric care sites in China from 17 May 2017 to 31 December 2021.

#### Inclusion criteria

The following inclusion criteria were used:

1. Age between 18 and 65 years
2. Diagnosis of MDD according to the Diagnostic and Statistical Manual of Mental Disorders, Fourth Edition (DSM-IV) criteria
3. Suitability for treatment with escitalopram or duloxetine
4. Total score of the QIDS-SR 16 ≥ 11 and total score of the 17-Item Hamilton Rating Scale for Depression (HAMD-17) ≥ 14
5. At least primary school level education, literacy, and ability to understand and complete questionnaires

#### Exclusion criteria

The exclusion criteria were as follows:

1. The presence of other psychiatric disorders(i.e., psychosis, schizophrenia, bipolar disorder, mania, hypomania, dementia, eating disorder)
2. Alcohol or other substance abuse problems
3. Item 3 of HAMD-17 (the suicide risk item) ≥ 3, indicating patients with serious suicide tendencies
4. Presence of serious physical disease
5. Women who were currently pregnant, planning pregnancy, or lactating
6. History of failing to tolerate or respond to escitalopram/duloxetine

#### Withdrawal from the acute phase (entrance to the follow-up phase)

This was caused due to the following reasons:

1. The presence of serious adverse events (SAEs) or the termination of current treatment due to adverse events (AEs)
2. Withdrawal of informed consent
3. Consideration that quitting will benefit the patient by the study investigators
4. Violation of research protocols
5. Switch to mania or hypomania (termination)

#### Withdrawal from the maintenance

This was caused based on the following reasons:

1. Lack of follow-up
2. Depression recurrence
3. Switch to mania, hypomania, or mixture
4. Suicide attempt, suicidal behavior, or self-injurious behavior
5. Hospitalized for mental illness

### Recruitment/consent procedures

This is a prospective, multicenter study conducted at 8 centers in China, 3 of which are psychiatric hospitals. The remaining 5 out of 8 centers are general hospitals, which are distributed in the south, east, north, and west of China. The Beijing Anding Hospital of the Capital Medical University will recruit 200 patients for this study. The remaining 700 patients will be recruited by the 7 following centers: First Affiliated Hospital of Kunming Medical University, Nanjing Brain Hospital Affiliated to Nanjing Medical University, Guangzhou Mental Health Research Center, Fourth Military Medical University of People’s Liberation Army, First Affiliated Hospital of Harbin Medical University, First Affiliated Hospital of Hebei Medical University, and West China Hospital of Sichuan University. Each center will recruit 100 subjects.

Patients will be mainly recruited via referral from psychiatrists in the outpatient departments. Once a potential participant is identified and meets the eligibility criteria, the investigating physician provides the patient and relatives written and oral information on the study in an understandable language and obtains written consent to take part in the study in line with the trial standard operating procedures. During this interview, the study characteristics will be explained, including the main objectives, potential benefits and adverse events, an explanation regarding the MBC and TAU, and the option to end their participation in the study at any time. At the screening visit, the trained research investigators (clinically experienced psychiatrists) and the raters collect the data and made judgments regarding eligibility. The Chinese version of the Mini-International Neuropsychiatric Interview (MINI) is used to confirm that the DSM-IV criteria for MDD are met and to assess the exclusion criteria. Fully informed consent is obtained from the patient, and the patient is not enrolled if s/he refuses or shows significant distress. The participants will be randomly assigned to either the MBC or the TAU group in a ratio of 1:1.

### Data management

All patient-reported questionnaires will be filled out by the patient in a paper format, and all data from the physiological measurements and clinical tests will be entered in a paper case report form. Subsequently, all data will be entered in EpiData (version 3.1 or newer) by the study personnel, using blinded double data entry to ensure data quality. The data entry form will support valid values and range checks where applicable. The original forms will be kept on file at a secure location on the study site for a period of 3 years after completion of the study. No data monitoring committee will be composed and no formal stopping guidelines and corresponding interim analyses are planned. No other interim analyses are planned.

### Randomization

Participants will be randomized to receive either the MBC or the TAU group in a 1:1 allocation ratio. The randomization code is created using a blocked randomization procedure in SAS 9.4 (SAS Institute Inc., Cary, NC, USA) by a statistician who is not involved in the study. The sequence and code used will be kept in an encrypted file accessible only to the trial statistician. Outcome assessors, trial participants, and care provider are unblind to the treatment allocation. The researchers are unblind to the treatment because they need to adjust the treatment based on the results of the score. Analyses are performed by blinded data analysts.

### Interventions and comparator

A total of 900 outpatients with MDD are enrolled and randomly assigned to the MBC and TAU groups, at a ratio of 2:1. They are subjected to the 12-week acute phase and 12-month maintenance-phase treatments.

#### Phase 1 (acute phase—12 weeks)

All subjects are administered either escitalopram (10 mg/day) or duloxetine (60 mg/day) in the initial treatment (the patients could lower the dose in case of increased side effects). The maximum dosage of drugs used in the acute phase was 20 mg/day (escitalopram) or 120 mg/day (duloxetine), and the dosage units are 5 mg and 20–30 mg, respectively. The acute phase treatment of the MBC groups includes dosage adjustments based on the scores of QIDS-SR 16 and the Frequency, Intensity, and Burden of Side Effects Rating Scale (FIBSER). The therapeutic decision is made at the baseline and at the 2-, 4-, 6-, and 8-week period. The patients with poor efficacy (QIDS-SR 16 > 9) could alter their drug treatment at the end of the 4th week (escitalopram or duloxetine). The adjustment of the drug type is performed only once and is completed within 1 week. During the visits, drug doses and treatment regimens are adjusted according to the severity of the adverse events (see Table 1 for specific dose adjustment).

**Table 1:** Treatment decision points and adjustments

Time	Assessment tool	Score	Escitalopram	Duloxetine
Baseline			Initial dose 10 mg/day; if the patient cannot tolerate, can reduce the dose.	Initial dose 60 mg/day; if the patient cannot tolerate, can reduce the dose.
Week 2	QIDS-SR 16 ≤ 5	Remission	Maintain current dose.	Maintain current dose.
	QIDS-SR 16 = 6–8	Partial response	Maintain the current dose or increase it to 15 mg/day	Maintain the current dose or increase it to 80/90 mg/day.
	Side effects cannot tolerate (item 3 of FIBSER = 5–6)		Maintain the current dose and symptomatic treatment of side effects/reduction of one dose unit.	Maintain the current dose and symptomatic treatment of side effects/reduction of one dose unit.
	QIDS-SR 16 ≥ 9	Treatment failure	Increase at least one dose unit or the drug has reached the highest dose to maintain the current dose.	Increase at least one dose unit or the drug has reached the highest dose to maintain the current dose.
	Side effects cannot tolerate (item 3 of FIBSER = 5–6)		Reduce a dose unit or switch to duloxetine.	Reduce a dose unit or switch to escitalopram.
Week 4	QIDS-SR 16 ≤ 5	Remission	Maintain current dose.	Maintain current dose.
	QIDS-SR 16 = 6–8	Partial response	Maintain the current dose or increase it to 20 mg/day.	Maintain the current dose or increase it to 120 mg/day.
	Side effects cannot tolerate (item 3 of FIBSER = 5–6)		Maintain the current dose and symptomatic treatment of side effects/reduction of one dose unit.	Maintain the current dose and symptomatic treatment of side effects/reduction of one dose unit.
	QIDS-SR 16 ≥ 9	Treatment failure	Patients who have been added to the highest dose and have completed 2 weeks may choose to change their medicine; if the maximum dose treatment is less than 2 weeks, maintain the current dose; for the dosage not reaching the maximum dose, increase it to 20 mg/day.	Patients who have been added to the highest dose and have completed 2 weeks may choose to change their medicine; if the maximum dose treatment is less than 2 weeks, maintain the current dose; for the dosage not reaching the maximum dose, increase it to 60 mg/day.
	Side effects cannot tolerate (item 3 of FIBSER = 5–6)		Reduce a dose unit or switch to duloxetine.	Reduce a dose unit or switch to escitalopram.
Weeks 6, 8, and 10	QIDS-SR 16 ≤ 5	Remission	Maintain current dose.	Maintain current dose.
	QIDS-SR 16 = 6–8	Partial response	Maintain the current dose or increase it to 20 mg/day.	Maintain the current dose or increase it to 120 mg/day.
	Side effects cannot tolerate (item 3 of FIBSER = 5–6)		Maintain the current dose and symptomatic treatment of side effects reduction of one dose unit.	Maintain the current dose and symptomatic treatment of side effects/reduction of one dose unit.
	QIDS-SR 16 ≥ 9	Treatment failure	The drug has been added to the highest dose to maintain the current dose; for the dosage not reaching the maximum dose, increase it to 20 mg/day.	The drug has been added to the highest dose to maintain the current dose; for the dosage not reaching the maximum dose, increase it to 120 mg/day.
	Side effects cannot tolerate (item 3 of FIBSER = 5–6)		Lower a dose unit or change the medicine.	Lower a dose unit or change the medicine.
Week 12	QIDS-SR 16 ≤ 5	Remission	Into the maintenance phase, randomized.	A. Comprehensive intervention group: maintenance drug treatment for 6 months and CCBT.
				B. Drug treatment group: maintenance drug treatment for at least 6 months.

*QIDS-SR 16* 16-item Quick Inventory of Depressive Symptomatology–Self-Report, *FIBSER* Frequency, Intensity, and Burden of Side Effects Rating Scale, *CCBT* computerized cognitive behavioral therapy

As a comparator, the TAU group is a traditional treatment based on clinical experience. The patients of the TAU group are treated by their psychiatrists at each outpatient visit, and their treatment drugs and adjustment are not limited.

At baseline and at weeks 2, 4, 6, 8, and 12 of the study, the patients complete self-evaluation reports. The observer evaluations are received at baseline and at weeks 4, 8, and 12 in both groups.

#### Phase 2 (maintenance phase—12 months)

The patients of the MBC treatment group who achieve response (QIDS-SR 16 score ≤ 9) in the acute phase were randomly assigned to the comprehensive intervention group or the drug treatment group for 6 months. The ratio was 1:1, and these patients are followed up for 12 months. The patients of the MBC and the TAU groups who exhibit poor treatment efficacy (QIDS-SR 16 > 9) and those who are terminated during the trial are followed up during the maintenance phase. The comprehensive intervention group maintains antidepressant treatment in the acute phase and computerized cognitive behavioral therapy (CCBT) for 6 months. The CCBT program included 20 units in total, and each unit requires approximately 30 min for completion. The patients receive feedback at the end of each unit. The drug treatment group includes continuing antidepressant treatment of the acute phase for 6 months, and no restriction is set on the treatment following 6 months. The maintenance phase assessments are performed at 1, 3, 6, 9, and 12 months and are completed by the researchers. In case of a termination event, subsequent treatment is provided by the research physician. The terminal events are defined as follows: [1] recurrence: evaluation of two consecutive QIDS-SR 16 ≥ 9 or diagnosis of MDD according to DSM-IV criteria [2]; hospitalization: hospitalized for mental illness [3]; switch to manic: hypomania, mania, mixed episodes according to DSM-IV criteria; and [4] self-injurious/suicidal behavior.

**Fig. 1:**
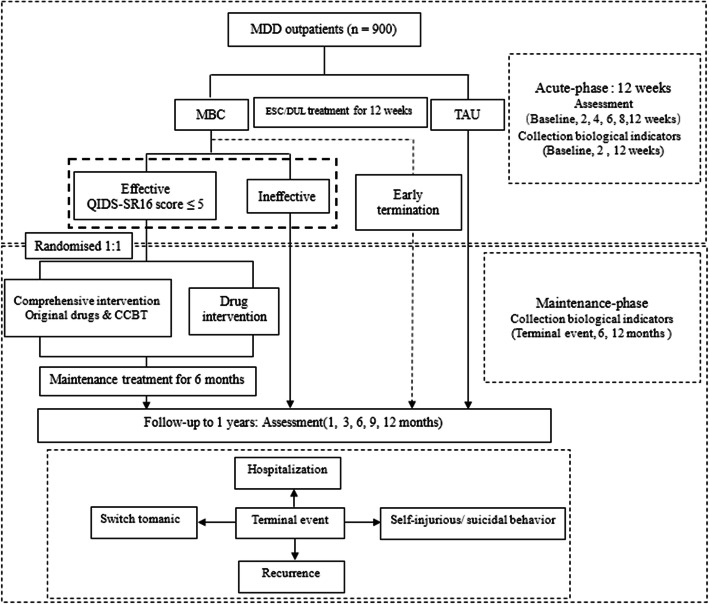
Overview of the study design. MDD, major depressive disorder; MBC, measurement-based care; TAU, treatment as usual; QRDS-SR 16, 16-item Quick Inventory of Depressive Symptomatology–Self-Report; CCBT, computerized cognitive behavioral therapy

See “Fig. 1 Research flow diagram” depicting a detailed overview of the research procedure.

### Interview time schedule

#### Acute phase treatment

A total of 7 visits (baseline, weeks 2, 4, 6, 8, 10, and 12) are conducted in the acute phase of the MBC treatment group, of which the visits performed at week 6 and week 10 can be performed by telephone interview. A total of 5 visits (baseline, weeks 2, 4, 8, and 12) are conducted in the TAU treatment group (a detailed overview is presented in Table 2). The telephone interview is conducted as follows: in case the patient fails to complete the interview within the specified time, the researcher performs a telephone interview to complete the questionnaire according to the items of the self-rating scale. All subjects complete self-assessment and mood mapping on the mobile app every day. In addition, they complete the evaluation of other scales according to the interview points.

**Table 2:** Overview of the acute phase interview

	Acute phase						
	0	Week 2, day 14 (± 3)	Week 4, day 28 (± 3)	Telephone interview (week 6)	Week 8, day 56 (± 3)	Telephone interview, week 10 (for patients who change drugs in week 4)	Week 12 (± 7)/early termination
Informed consent	×						
Inclusion/exclusion criteria	×						
General data	×						
Medical history and mental symptoms	×						
MINI	×						
Somatic and neurological examination	×						
Vital signs/weight	×		×		×		
QIDS-SR 16	×	×	×	×	×	×	×
FIBSER	×	×	×	×	×	×	×
PDQ-9	×		×		×		×
SDS	×		×		×		×
FBS	×		×		×		×
Q-LES-Q-SF	×		×		×		×
HAMD-17	×		×		×		×
YMRS	×		×		×		×
Acceptability/feasibility		×	×		×		×
Adverse reactions	×	×	×	×	×		×
Biological sample collection	×	×					×

*MINI* Mini-International Neuropsychiatric Interview, *QIDS-SR 16* 16-item Quick Inventory of Depressive Symptomatology–Self-Report, *FIBSER* Frequency, Intensity, and Burden of Side Effects Rating scale, *PDQ-9* Patient Health Questionnaire-9, *SDS* Self-Rating Depression Scale, FBS Family Burden Scale of Disease, *Q-LES-Q-SF* Quality of Life Enjoyment and Satisfaction Questionnaire–Short Form, *HAMD-17* 17-Item Hamilton Rating Scale for Depression, *YMRS* Young Mania Rating Scale

#### Maintenance phase treatment

Two groups are interviewed at the end of months 1, 3, 6, 9, and 12 during the follow-up period. The subjects finish all self-assessment scales and all observer rating scales.

### Drug/therapy combination

During the acute phase, the MBC group is only allowed to use research drugs, the TAU group is not limited with drugs. During the maintenance phase, the combined use of drugs is not restricted in the two groups.

Benzodiazepines or non-benzodiazepines are used for the treatment of insomnia, whereas benzodiazepines or buspirone and tandospirone are used for the treatment of anxiety. The type, dosage, and time of drug treatment use are recorded. The use of drugs for somatic diseases is allowed, and the type and dosage of drugs are recorded. Any physical therapy or systemic psychotherapy is prohibited during treatment.

### Protocol amendments

Decisions regarding protocol amendments will be taken by the study core team encompassing the coordinating investigator, trial coordinator, trial statistician, medical coordinator, and data management. Any significant amendments to the protocol or supporting materials will be submitted to the Beijing Anding Hospital ethics review committee, Beijing Municipal Commission of Health and Construction for their approval. Records of relevant communication with the Research Ethics Committee and the regulatory authorities are kept by the coordinating investigator. Furthermore, after protocol amendments or upon relevant updates during the study, a newsletter will be sent to all participating centers.

### Quality control

Each clinical site contained one research coordinator responsible for the coordination of the site in addition to the principal investigator, 2 to 4 investigators (clinically experienced psychiatrists) responsible for recruitment, clinical evaluation, or blood specimen collection and one member of the staff responsible for entering paper case report forms (CRFs) into EDC. In order to improve the adherence of the subjects to the protocol, a person is assigned to follow up the appointment and provided convenient services to the subjects.

The quality management plan is elaborated prior to the study initiation. A clinical research assistant (CRA) or a medical monitor is assigned by the study team and is responsible for supervising the compliance to the study protocol and checking the conformance of the paper-based and the web-based CRF. In addition to the quality monitoring performed by the CRA or the medical monitor, the data manager would also issue regular queries through EDC.

The executive management team organizes all principal investigators and/or research coordinators monthly via email or WeChat in order to discuss the difficulties and share the experience of ensuring the protocol implementation. In addition, a group of experts assigned by the executive management team visits each site once a year to guide the investigator team, confirm protocol implementation, and assess the reliability of the clinical evaluation.

The ethics committee must review and harmonize the protocol and informed consent prior to the inclusion of any subject. Prior to the implementation of any program, the subject must sign and sign the informed consent form approved by the ethics committee. The outpatient doctors recommended suitable depression patients, and the study doctors were responsible for introducing relevant contents and signing informed consent forms, and one informed consent form was handed to the enrolled patients. Patients have the right to withdraw from the study and continue treatment at any time. Patients can contact the competent doctor at any time if any adverse events occur during the study. The subject’s personal information will be kept confidential.

### Outcomes

Efficacy and acceptability are compared between the OMBC and the TAU groups.

1. The primary outcomes of the study are the complete remission rate (proportion of patients with QIDS-SR 16 total score ≤ 5) of the MBC and TAU groups at the end of the acute phase and the comparison of the recurrence rate and recurrence time in 1 year between the two groups (recurrence: evaluation of two consecutive QIDS-SR 16 score ≥ 9 or diagnosis of MDD according to DSM-IV criteria).
2. The secondary outcomes included the comparison of the changes caused on QIDS-SR 16, Patient Health Questionnaire-9 (PHQ-9), and HAMD-17 from the baseline as well as the response rate (response: QIDS-SR 16 score ≤ 8; reduction of HAMD-17 ≥ 50%) between the two groups at the end of the acute phase. Moreover, additional parameters are measured including the comparison of the recurrence rate between the two groups in the maintenance phase, the incidence of other terminal events and adverse reactions, social function assessment [Self-Rating Depression Scale (SDS), Family Burden Scale of Disease (FBS), and Quality of Life Enjoyment and Satisfaction Questionnaire–Short Form (Q-LES-Q-SF)], and the acceptability, compliance, and feasibility of the MBC treatment mode.

### Assessment tools

#### Diagnostic tools

The MINI [20] is used as the structured diagnostic interview instrument in this study.

#### Self-assessment scales

The QIDS-SR is a self-assessment, which rates depression symptoms [21].

The FIBSER Questionnaire uses 3 questions with a 6-point Likert measurement scale [22]. This tool measures 3 side effect domains of impact, such as frequency, intensity, and burden.

The PHQ-9 is a multipurpose instrument for screening, diagnosing, monitoring, and measuring the severity of depression [23].

The SDS is a 20-item self-report questionnaire that is widely used as a screening tool, covering affective, psychological, and somatic symptoms associated with depression [24].

The FBS is used to assess the family burden and contains a total of 24 items that involve the following 6 factors: economic burden, impact on daily activities, impact on social life, impact on free time, impact on physical health, and impact on mental health [25].

The Q-LES-Q-SF is a self-report measure designed to enable investigators to easily obtain sensitive measures of the degree of enjoyment and satisfaction experienced by subjects in various areas of daily functioning [26].

#### Observer-rating scales

The Chinese version of the HAMD-17 has satisfactory psychometric properties in terms of validity and reliability. The internal reliability and the validity are good (Cronbach alpha = 0.714; *r* = − 0.487) [27].

The Young Mania Rating Scale (YMRS) [28] is one of the most frequently utilized rating scales to assess manic symptoms. The scale has 11 items and is based on the patient’s subjective report of his or her clinical condition over the past 48 h.

#### Evaluation of MBC treatment mode

The acceptability (1–5 points: 1 very bad, 2 relatively bad, 3 average, 4 relatively good, 5 very good) and feasibility (1–4 points: 1 completely unfeasible, 2 not very feasible, 3 moderately feasible, 4 very feasible) were evaluated by the psychiatrist on weeks 4, 8, 12, and 16 or at early withdrawal. The acceptability was evaluated by the patients.

### Sample collection

Biological sample collection including blood, urine, and feces was only available in certain centers. The samples are collected at baseline and at weeks 2 and 12 in the acute phase. Subsequently, they are collected every 3 months during the follow-up period and at the end of the terminal events. Biological sample collection is only applicable to subjects who have been approved by the ethics committee of the sub-center and have signed the informed consent form.

### Sample size

#### Acute phase

According to previous research [19, 29], the response rate of the TAU group was 32–44.3% (assuming that the MBC group was increased by 10%). The ratio between the MBC and the TAU groups was 2:1 (*α* = 0.05, and power = 80%). At least 462 and 231 subjects were required for the MBC and the TAU groups, respectively. Considering that 20% was missing data, 600 and 300 subjects were required for these two groups.

#### Maintenance phase

According to previous research, the cumulative annual recurrence rate of the depressive disorder was 42%. The HR in the comprehensive intervention group was 3 and reduced the recurrence rate by 5% (*α* = 0.05 and power = 80%). At least 100 subjects were required for each group. Considering that 20% of the data were missing, 125 subjects were included in each group. Since the subjects of the second stage originated from the effective subjects of the MBC group, it was considered that the MBC group was able to meet the requirements of the second stage sample by assuming that 80% of the patients were effectively treated (more than 260 subjects). In summary, a total of 900 subjects were required in the present study.

### Statistical analysis

Efficacy evaluations are performed on the full analysis set (FAS) and on the per-protocol set (PPS) datasets. FAS followed the intent-to-treat (ITT) principle. The subjects are analyzed according to the treatment that they have been assigned randomly. The PPS dataset includes all subjects in the FAS who receive any study medication and exhibit no serious protocol deviations. The safety set (SS) dataset includes all subjects who received any amount of study drug and have at least one post-baseline safety assessment.

All collected data are analyzed using the SAS Statistical Package (version 9.4; SAS Institute Inc., Cary, NC, USA). The data distribution is tested for normality using the Shapiro-Wilk test, and the differences between the groups are selected according to the distribution of the variables (*t* test/*F* test). The variables that are not normally distributed are tested by the non-parametric test (rank sum test). The continuous variables are presented as mean ± standard deviation. The chi-squared test is employed for percentages of variables.

The scores of QIDS-SR16, PHQ-9, and HAMD-17 in the two groups at the end of the acute phase are compared with the baseline levels by the paired *t*-test, and the covariance analysis is used for comparison between the two groups. The chi-squared test is used to compare the remission rate of the acute phase and the recurrence rate during the maintenance phase. The comparison of the remission rates between the two groups at the acute phase is based on the analysis of the mixed-effect model for the repeated measures (MMRM) model and the missing values are not imputed. Kaplan–Meier survival analysis is used to compare the recurrence time between the comprehensive intervention group and the drug treatment group within 1 year. The Cox proportional hazard regression model is applied to compare the estimated time to recurrence between the two groups while controlling for covariates. A *P* value lower than 0.05 (*P* < 0.05) is considered for significant differences.

### Monitoring

AE is an adverse medical condition that occurs after the use of the drug by the subjects. AE may not be related to the treatment. The correlation between AE and the treatment was evaluated by the researchers. Following the signing of the informed consent form, any serious adverse events that occurred during the research are reported by the ethics committee and by the hospital management department of the sub-center within 1 working day after the researcher is informed. The researchers completed the SAE report form and send it to the person in charge of the SAE. The SAE report is filed with the ethics committee of the leading unit (Beijing Anding Hospital Affiliated to Capital Medical University).

## Discussion

It is well known that the recognition and treatment rates of MDD remain very low. Although we have proved that MBC is a feasible and more effective method than TAU for patients with moderate depression to MDD, further studies need to be performed to optimize this method. The present study included a 12-week, randomized control trial of acute phase treatment and a 1-year maintenance phase treatment. It aimed to optimize the existing index system, evaluate the model and decision-making process of the MBC treatment, and form a standardized quantitative treatment technology suitable for MDD. The optimization of MBC relies mainly on establishing a comprehensive MBC framework based on continuous assessment that will contribute to decrease the relapse or recurrence rate of depression. The present study can provide further guidance for the standardized diagnosis and treatment of depression. By conducting this study, we hope to lay a foundation for patient disease self-management in the future.

### Strengths and limitations of this study

A strength is that the OMBC treatment protocol—optimization of MBC treatment for depression, establishes a comprehensive MBC framework based on all-round, continuous assessment for depression.

To our knowledge, this will be the first study to evaluate OMBC in China. This study is a multi-center study with a large sample size.

However, study design limitations are that long follow-up up to 12 months resulted in a high drop-out rates, which has a certain impact on the results of the study.

In this study, only patients with unipolar depression were included, which could potentially influence further application.

Antidepressants are limited to citalopram and duloxetine, which will limit the enrollment of the patients and affect the expansion of results.

### Current trial status

Protocol version number (last updated): 3.0. Protocol version date (last updated): 11 October 2018.

Participant recruitment for this randomized clinical trial began on 17 May 2017 and is to be completed by 31 December 2021.

Due to the global outbreak of COVID-19, the overall enrollment of the program has been delayed, the program has been postponed, and the follow-up of patients has not yet been completed (April 2022). Follow-up of the last patients will be completed by the end of 2022. Factors such as inappropriate selection of journals, a long review period, the long time to write the protocol and polish the language, and other factors contributed to the long submission cycle of our protocol.

“The protocol should have been submitted prior to the end of recruitment; if not, the authors should give reasons for the delay.” *Trials* advises that study protocols are submitted well before recruitment completes; however, we will also on occasion consider study protocols submitted before the last patient/last visit.

## Data Availability

The final trial data for this protocol can be supplied on request. The datasets analyzed during the current study and statistical code are available from the corresponding author on reasonable request, as is the full protocol.

## Abbreviations

MBC: Measurement-based care
OMBC: Optimization of measurement-based care
TAU: Treatment as usual
MDD: Major depressive disorder
QIDS-SR 16: 16-item Quick Inventory of Depressive Symptomatology–Self-Report
PHQ-9: Patient Health Questionnaire-9
HAMD-17: 17-item Hamilton Rating Scale for Depression
APA: American Psychiatric Association
DSM-IV: Diagnostic and Statistical Manual of Mental Disorders, Fourth Edition criteria
FIBSER: Burden of Side Effects Rating scale
CCBT: Computerized cognitive behavioral therapy
CRF: Case report form
EDC: Electronic Data Capture System
CRA: Clinical research assistant
SDS: Self-Rating Depression Scale
FBS: Family Burden Scale of Disease
Q-LES-Q-SF: Quality of Life Enjoyment and Satisfaction Questionnaire–Short Form
MINI: Mini-International Neuropsychiatric Interview
YMRS: Young Mania Rating Scale
FAS: Full analysis set
PPS: Per-protocol set
ITT: Intent-to-treat
SS: Safety set
LOCF: Last-observation-carried-forward
ROC: Receiver operating characteristic
AUC: Area under the curve
AE: Adverse event
SAE: Serious adverse event
